# NIR‐Activated Polydopamine‐Coated Carrier‐Free “Nanobomb” for In Situ On‐Demand Drug Release

**DOI:** 10.1002/advs.201800155

**Published:** 2018-05-09

**Authors:** Minghui Li, Xuetan Sun, Ning Zhang, Wei Wang, Yang Yang, Huizhen Jia, Wenguang Liu

**Affiliations:** ^1^ School of Materials Science and Engineering Tianjin Key Laboratory of Composite and Functional Materials Tianjin University Tianjin 300350 P. R. China; ^2^ State Key Laboratory of Molecular Engineering of Polymers Fudan University Shanghai 200433 P. R. China

**Keywords:** carrier‐free nanoparticles, chemo‐/photothermal therapy, drug delivery, NIR‐responsive materials, polydopamine films

## Abstract

Carrier‐free nanoparticles with high drug loading have attracted increasing attention; however, in situ on‐demand drug release remains a challenge. Here, a novel near‐infrared (NIR) laser‐induced blasting carrier‐free nanodrug delivery system is designed and fabricated by coating doxorubicin (DOX) nanoparticles (DNPs) with a polydopamine film (PDA) that would prolong the blood circulation time of DNPs and avoid the preleakage of the DOX during blood circulation. Meanwhile, the NH_4_HCO_3_ is introduced to trigger in situ “bomb‐like” release of DOX for the production of carbon dioxide (CO_2_) and ammonia (NH_3_) gases driven by NIR irradiated photothermal effect of PDA. Both in vitro and in vivo studies demonstrate that the carrier‐free nanovectors with high drug loading efficiency (85.8%) prolong tumor accumulation, enhance chemotherapy, achieve the synergistic treatment of chemotherapy and photothermal treatment, and do not induce any foreign‐body reaction over a three‐week implantation. Hence, the delicate design opens a self‐assembly path to develop PDA‐based NIR‐responsive multifunctional carrier‐free nanoparticles for tumor therapy.

## 1 Introduction

Nanocarrier as delivery systems for therapeutic agents have shown enormous potential in cancer therapy.^1^, ^2^ There are numerous smart multifunctional nanosystems that have been designed based on the characteristics of tumors.^3^, ^4^, ^5^, ^6^, ^7^, ^8^ However, their applications still suffer from the drawbacks of the lower drug loading efficiency and/or the low drug effective concentration in the site of disease. An ideal nanoparticle is required to have a high drug loading efficiency, long circulation time, and the encapsulated cargos can be rapidly released in the tumor cells to achieve the interaction with biological targets. So far, attempts have been made to design a variety of functional nanocarriers. Fattahi et al. have used electrojetting technique to produce 1,3‐bis(2‐chloroethyl)‐1‐nitrosourea (BCNU)‐loaded poly(lactic‐*co*‐glycolic acid)(PLGA) microcapsules with higher drug encapsulation efficiency; however, currently available nanoparticles are still difficult to fulfill all the design requirements.^9^, ^10^, ^11^, ^12^, ^13^, ^14^, ^15^

Carrier‐free nanodrugs have been reported to improve the drug loading efficiency and thus avoid the side effects of inert materials introduced. Recently, Zhang and co‐workers used pure doxorubicin (DOX) nanoparticles to fabricate a chemotherapy system, where the drug payload reached as high as 90.47%.^16^ Lee and co‐workers, produced self‐carried curcumin nanoparticles with drug loading capacities > 78 wt%, which was much higher than that of the traditional drug delivery systems with drug loading capacities (<10%).^17^ To achieve better biocompatibility, the polyethylene glycol (PEG) was frequently introduced through hydrophobic interactions. However, the drug release rate was also too high, and it was very detrimental to the drug cycle, which could cause unsatisfactory side effects for the leakage of drugs in advance, eventually leading to a very low drug concentration in lesion site. Thus, ultimate tumor treatment efficiency was severely limited. Developing drug delivery systems simultaneously including high drug loading efficiency and high drug accumulation concentration at the lesion site would be beneficial for in vivo applications.

Dopamine, as a neurotransmitter, was widely distributed in human. Recent studies reported that the dopamine could self‐polymerize to form surface‐adherent polydopamine (PDA) films onto a wide range of materials either organic or inorganic by a spontaneous oxidation reaction in an alkaline solution (pH = 8.5).^18^, ^19^, ^20^, ^21^ It was widely used as material surface modification since the PDA shells are stable enough to reach the target cells after intravenous injection.^22^, ^23^ Previous study indicated that the PDA film was highly superior for in vivo photothermal therapy: it showed biodegradability, a high median lethal dose, and did not induce long‐term toxicity during their retention in rats.^24^ Coincidentally, ammonium bicarbonate (NH_4_HCO_3_) can be thermally triggered to generate carbon dioxide (CO_2_) and ammonia (NH_3_) gases, which are normal metabolites of the human body, and can be completely eliminated from living organisms without causing toxicity to humans.^25^, ^26^, ^27^ Recently, this nontoxic substance has been widely used to facilitate the release of loaded cargoes by encapsulation into liposome, PLGA nanoparticles, and so on.^28^, ^29^, ^30^, ^31^

**Figure 1:**
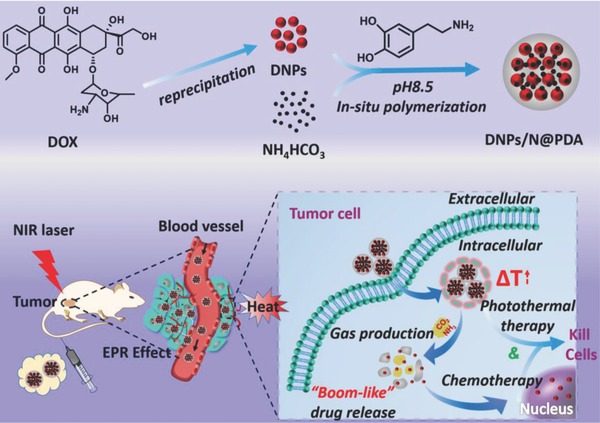
Preparation of polydopamine‐coated and NIR‐responsive carrier‐free “Nanobomb” (DNPs/N@PDA) based on packing the DNPs and NH_4_HCO_3_ with polydopamine (PDA) and schematic illustration of the stable blood circulation of DNPs/N@PDA and on demand “bomb‐like” drug release and enhanced chemo‐/photothermal therapy triggered by NIR irradiation.

In this work, we fabricated a NIR‐responsive polydopamine coated carrier‐free "nanobomb” for in situ on‐demand drug release. As shown in **Figure** **1**, the hydrophobic chemotherapeutic (DOX) was used to self‐assembly into carrier‐free nanodrugs, referred to DNPs. Then a thin PDA film was synthesized and contacted onto the surface of the coassembly of DNPs and NH_4_HCO_3_ (denoted as DNPs/N@PDA). We hypothesize that the circulation time of DNPs can be prolonged and the preleakage of the DOX can be avoided for the protection of PDA film, thus facilitating the passive tumor accumulation of DOX through the well‐established enhanced permeability and retention (EPR) effect. After transporting to tumor regions, the PDA films can be broken by the CO_2_ and NH_3_ produced from the photothermal decomposition of NH_4_HCO_3_ under NIR irradiation, and the loaded DNPs is released in a “bomb‐like” manner due to the strong near‐infrared absorption and high photothermal conversion efficiency (40%) of PDA film.^32^, ^33^ In principle, this transition would make DNPs inclined to aggregate and thus slow down reentry into the blood stream because of its inherently hydrophobic nature, which consequently leads to a locally long‐term entrapment of drug‐vehicle system within neighboring tumor cells. And, the photothermal effect of PDA can trigger the release of small DNPs (≈5 nm) that enable deep and uniform penetration into more cancer cells, ultimately enhancing the chemotherapy of the DOX.^34^, ^35^ Attractively, the structure alteration will simultaneously induce a selectively fast drug release, favoring the enhanced drug efficacy inside tumor cells and finally realizing the chemo‐/photothermal synergistic therapy of cancer. At last but not least, some other functional modules, e.g., targeting moieties and/or imaging probes can be introduced into the surface of the PDA film for the existence of functional groups of catechol and amine.^10^, ^18^, ^36^ As such, it therefore offers a new avenue for fabrication of the next generation of multifunctional cancer drug delivery systems with high drug loading efficiency and high accumulation tumor concentration to achieve the site and time dual‐controlled drug delivery for tumor therapy.

## 2 Results and Discussion

### 2.1 Fabrication and Characterization

The synthetic process of polydopamine‐coated carrier‐free near‐infrared (NIR) laser‐induced “bomb‐like” nanoplatform was illustrated in Figure 1. The carrier‐free nanodrugs were prepared by reprecipitation method. First, the preprepared DOX– dimethyl sulfoxide (DMSO) solution was injected dropwise into the deionized water under vigorous stirring. For the sudden change in the solvent environment, the DOX molecules could aggregate and precipitate to form DOX NPs (termed DNPs).^16^ Thereafter, PDA coating was introduced into the DNPs nanosystems containing NH_4_HCO_3_ via an oxidative self‐polymerization of dopamine under a weakly alkaline solution and shaking at room temperature overnight based on the fact that the PDA film can attach onto the surface of a wide range of materials including organic and inorganic.^19^ The final “bomb‐like” nanosystem was synthesized and defined as DNPs/N@PDA. With the same method, the PDA film‐coated DNPs nanosystems without NH_4_HCO_3_ was prepared and defined as DNPs@PDA. The PDA film also contributed to the outstanding photothermal ability of this nanosystem, which will be described later on.

**Figure 2:**
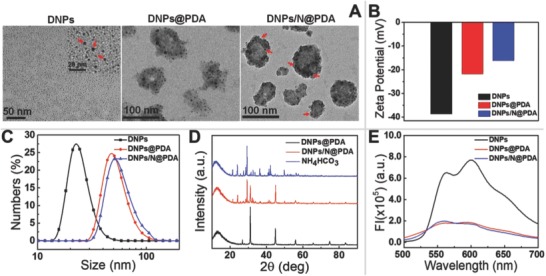
A) TEM images of DNPs, DNPs@PDA, DNPs/N@PDA (DNPs were marked with red arrow). B) Zeta potentials of the DNPs, DNPs@PDA, and DNPs/N@PDA. C) DLS profiles of DNPs, DNPs@PDA, and DNPs/N@PDA. D) XRD patterns of NH_4_HCO_3_, DNPs@PDA, and DNPs/N@PDA. E) Fluorescence spectra of DNPs, DNPs@PDA, and DNPs/N@PDA.

In order to evaluate the morphology of the DNPs/N@PDA NPs, transmission electron microscopy (TEM) was used. As shown in **Figure** **2**A, DNPs displayed a spherical and uniform morphology with a diameter of around 5 nm. After introduction of NH_4_HCO_3_ and being coated by PDA, the diameter of DNPs became bigger and the PDA film served as superficial coverage. The image of the DNPs/N@PDA revealed an average size of about 70 nm, which was slightly smaller than that measured by dynamic light scattering (DLS) (Figure 2C) for the shrinkage of nanoparticles in a drying state during TEM sample preparation. The TEM images of DNPs@PDA and DNPs/N@PDA in Figure 2A clearly indicated that the nanoparticles consisted of a lot of small spherical domains. Thus, the as‐prepared nanoparticles were formed through the secondary aggregation of small nanoparticles from DNPs via surface‐deposited PDA.^21^ The zeta potential was measured to investigate the surface modification and charge changes of the DNPs (Figure 2B). Bare DNPs showed a zeta potential of −37.5 ± 0.45 mV, and after PDA coating, the zeta potential increased to −15.0 ± 0.45 mV, which might be ascribed to the presence of hydroxyl of polydopamine.^10^ The DLS measurements at different time intervals and the study of protein bovine serum albumin (BSA) adsorption demonstrated that DNPs/N@PDA dispersion exhibited high stability under 10% serum‐containing conditions (Figure S1, Supporting Information). It reported that the fluorescence of the dye can be quenched effectively by the PDA. In this work, it came out that the fluorescence intensities of DNPs were sharply decreased after PDA coating (Figure 2E). Taken together, our observations indicated that the PDA was well coated on the surface of DNPs. Additionally, X‐ray diffraction (XRD) patterns (Figure 2D) revealed that NH_4_HCO_3_ as a drug release promoter was successfully entrapped in the nanoparticles.

### 2.2 In Vitro Photothermal Effect and Drug Release Profiles

**Figure 3:**
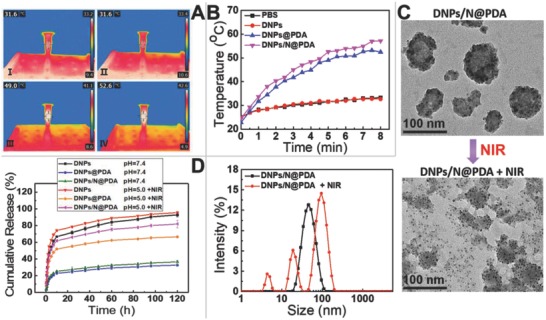
A) Infrared thermographic images at 5 min of NIR laser irradiation (808 nm, 5 W cm^−2^), (A‐I) PBS, (A‐II) DNPs, (A‐III) DNPs@PDA, (A‐IV) DNPs/N@PDA. B) Temperature elevation curves of PBS, DNPs, DNPs@PDA, and DNPs/N@PDA with NIR laser irradiation of 808 nm at a power density of 5 W cm^−2^ for 8 min. C) TEM images of DNPs/N@PDA and NIR‐irradiated DNPs/N@PDA, and the hydrodynamic diameters of DNPs/N@PDA before and after NIR laser irradiation (808 nm, 5 W cm^−2^) for 5 min. D) Cumulative release profiles of DOX from DNPs, DNPs@PDA, and DNPs/N@PDA in PBS with different pHs without or with NIR irradiation (808 nm, 5 W cm^−2^, 5 min).

An important feature of DNPs/N@PDA is their NIR light‐induced thermal effect, which could be used to induce the decomposition of NH_4_HCO_3_ and further resulted in the sharp release of the loaded drugs for the ultimate photothermal therapy. While, as a drug delivery system for cancer therapy, the drug loading efficiency and rapid intracellular release are critical. Thus, the drug loading efficiency of DOX was determined by fluorescence spectrometry; it was estimated to around 85.8% (Figure S2, Supporting Information). The photothermal conversion experiments were performed to study the photothermal effect induced by NIR irradiation. As shown in **Figure** **3**A,B, the DNPs@PDA and DNPs/N@PDA exhibited similar temperature rising profiles. The temperatures of DNPs@PDA and DNPs/N@PDA aqueous solution (0.1 mg mL^−1^) increased rapidly and the maximal temperatures at 5 min increased to 49.0 and 52.6 °C, respectively. In comparison, PBS and the DNPs solution (0.1 mg mL^−1^) showed little temperature change and the maximal temperature of PBS and DNPs solution only increased to 31.6 °C under the same laser irradiation (808 nm, 5 W cm^−2^, 5 min). Such excellent photothermal conversion effect of DNPs/N@PDA was mainly attributed to the coating of PDA. Therefore, DNPs/N@PDA had a high photothermal effect and was favorable for photothermal therapy.

We next investigated the NIR‐activated “bomb‐like” effect of the DNPs/N@PDA nanoparticles because the NH_4_HCO_3_ encapsulated in the nanoparticles could produce CO_2_ and NH_3_ gases for the photoheat of the PDA films under NIR laser irradiation. Indeed, after NIR laser irradiation, the size of many DNPs/N@PDA nanoparticles increased and new signals representing smaller particles of DNPs emerged (Figure 3C), indicating the gas generation from ammonium bicarbonate encapsulated in the DNPs/N@PDA nanoparticles was responsible for the observed change of size and morphology after NIR laser irradiation. The drug release profile of DNPs, DNPs@PDA, and DNPs/N@PDA was further evaluated under diverse stimuli such as pH and NIR light. As shown in Figure 3D, for DNPs, a burst release was observed and about 60% DOX was released during 12 h in pH 7.4 or 5.0 (simulate the normal physiological environment, including the conditions during nanoparticle transport in blood, and the acidic environment of tumors, respectively); while, after PDA coating, no burst release took place at pH 7.4 and less than 30% DOX leaked out across 120 h period, indicating the PDA coverage inhibited the preleakage of DOX, meanwhile enhancing the stability of DNPs in the physiological condition. Upon exposure to lower pH environment (pH = 5.0), the DNPs/N@PDA showed a relatively fast release of DOX molecules, with about 56.4% of the releasing over the same period (Figure S3, Supporting Information). The enhanced drug‐releasing rate at lower pH should be mainly ascribed to the pH sensitivity of PDA coating and NH_4_HCO_3_.^37^, ^38^, ^39^, ^40^ More attractively, when exposed to a momentary NIR irradiation (808 nm, 5 W cm^−2^, 5 min), the drug release rate was significantly enhanced and showed a similar release profile as DNPs. The release profiles of DNPs with or without NIR irradiation were almost the same. These results showed that the NIR‐induced drug‐release could be attributed to the local increased temperature owing to the high photothermal conversion efficiency of PDA film, which resulted in the decomposition of NH_4_HCO_3_ and facilitated the diffusion of DOX into the solution. This was supported by the size data and TEM images in Figure 3C. We noted that the fluorescence intensity of DNPs/N@PDA returned to the same level as the DNPs (Figure S4, Supporting Information) after NIR irradiation (808 nm, 5 min at 5 W cm^−2^). All the results demonstrated that NIR laser irradiation could boost the DOX release by opening the PDA films coated on the nanodrug itself.

### 2.3 In Vitro Cellular Uptake and Cytotoxicity Assay

**Figure 4:**
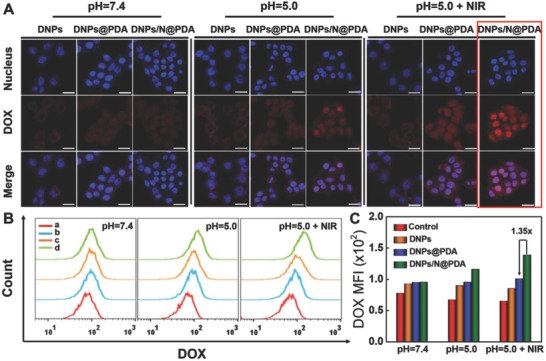
A) Confocal fluorescence images of HeLa cells exposed to DNPs, DNPs@PDA, and DNPs/N@PDA for 4 h at pH 7.4, pH 5.0, and pH 5.0 + NIR, respectively. Scale bars: 25 µm. B) Flow cytometric profiles of HeLa cells after treated by different samples for 4 h at pH 7.4, pH 5.0, and pH 5.0 + NIR, respectively. The concentration of DOX was fixed at 5 µg mL^−1^. a: negative control, b: DNPs, c: DNPs@PDA and d: DNPs/N@PDA. C) Quantification analysis of DOX fluorescence intensity in HeLa cells after incubation for 4 h at pH 7.4, pH 5.0, and pH 5.0 + NIR, respectively. NIR irradiation: 808 nm, 5 min at 5 W cm^−2^.

Efficient transport into cells is one of the main factors affecting the efficacy of DOX. After 4 h coincubation with different formulations including DNPs, DNPs@PDA, and DNPs/N@PDA, HeLa cells were photographed using confocal laser scanning microscopy, offering a visual inspection of cell entry. As can be seen in **Figure** **4**, at 4 h after cells being treated with DNPs, the fluorescence was observed in the cytoplasm, while very little was observed in the nuclei. In comparison, after 4 h incubation of cells with DNPs@PDA and DNPs/N@PDA, relatively intense fluorescence could be observed in the cytoplasm and meanwhile a small amount of fluorescence was found in the nuclei. When the cells were cultured with the different formulations at pH 5.0, the red fluorescence from DNPs was also observed in the cytoplasm, while the red fluorescence from DNPs@PDA and DNPs/N@PDA was mainly observed in the nuclei. The fluorescence intensity of DOX in HeLa cells treated by PDA coated nanoparticles at pH 5.0 was approximated to 1.16–1.40 folds stronger than that at pH 7.4. The fluorescence intensity of DOX in the groups treated by DNPs was nearly the same either at pH 7.4 or 5.0. That may be because that the PDA film was pH sensitive, and it would shed and broke up in acidic conditions; thus the drug was concentrated in lysosomes and resulted in the released DOX diffusion from cytoplasm to the nuclei.^38^ These experiments demonstrated that PDA coated nanoparticles could be ingested by HeLa cells and internalized into the cytoplasm. Upon NIR laser irradiation at pH 5.0, the fluorescent signals of DOX were in the nucleus domain when the cells were coincubated with the PDA containing nanosystems especially with the DNPs/N@PDA. The fluorescence intensity of DOX in HeLa cells treated by DNPs/N@PDA plus NIR irradiation was approximated to 1.4 folds stronger than that in the cells treated by DNPs@PDA plus NIR irradiation as well as 1.6 folds stronger than that in the cells treated by DNPs either with or without NIR light. This further confirmed the rapid release of DOX from the lysosome for the fast breaking of PDA film, which resulted from the thermal decomposition of NH_4_HCO_3_ under NIR irradiation, consequently leading to the death of the cancer cells. Taken together, the PDA film of DNPs@PDA and DNPs/N@PDA shed after accumulating in the lysosomes and could also be broken quickly by the CO_2_ and NH_3_ produced from the NH_4_HCO_3_ encapsulated into the “nanobomb” after treatment by NIR laser, and a novel lysosome and NIR double‐responsive strategy potential for effective cancer therapy was proposed in our case of DNPs/N@PDA.

**Figure 5:**
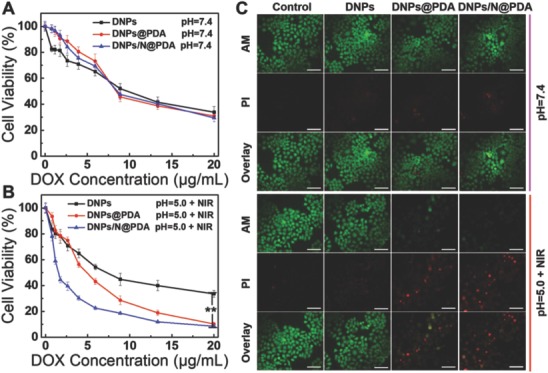
A,B) In vitro cytotoxicity of DNPs, DNPs@PDA, and DNPs/N@PDA at pH 7.4 and pH 5.0 with NIR laser irradiation (808 nm) of 5 W cm^−2^ for 1 min at different DOX concentrations on HeLa cells after 48 h incubation (**p < 0.01 versus DNPs/N@PDA group with NIR irradiation). C) Confocal fluorescence microscopy images of HeLa cells of different treatments for 48 h costained with Calcein‐AM (green, live cells) and propidium iodide (PI) (red, dead cells) before and after laser illumination (808 nm, 5 W cm^−2^, 1 min). The DOX concentration was fixed at 5 µg mL^−1^. Scale bars: 100 µm.

To verify our expectations, the cytotoxicity of DNPs, DNPs@PDA, and DNPs/N@PDA with HeLa cells was measured by 3‐(4,5‐Dimethyl‐2‐thiazoyl)‐2,5‐diphenyl tetrazolium bromide (MTT) assay. The cells were incubated with the different samples for 4 h; after that, the medium was replaced by fresh medium and suffered to NIR laser irradiation (808 nm, 5 W cm^−2^) for 1 min. As shown in the **Figure** **5**A, the viability of cells treated with DNPs@PDA plus NIR containing 4 µg mL^−1^ of DOX at pH 5.0 decreased to 56%, while DNPs/N@PDA plus NIR containing the same concentration of DOX induced up to 70% cell death after coincubation for 4 h due to the DOX “bomb‐like” release for the broken of PDA film resulted from the thermal decomposition of NH_4_HCO_3_ under NIR laser irradiation as proved above (Figure 3C; Figure S3, Supporting Information). In the meantime, the cell viability of DNPs, DNPs@PDA, and DNPs/N@PDA was respectively measured to be 34%, 31%, and 30% at pH 7.4; 30%, 20%, and 15% at pH 5.0 after coincubation with HeLa cells for 48 h without NIR irradiation (Figure S5A, Supporting Information). The growth tendency of the cells was almost the same with that of the cells treated by NIR laser (Figure 5B). There was no obvious difference in the DNPs‐treated groups with or without irradiation even at pH 7.4 or 5.0, revealing that the NIR laser alone could not cause an evident effect on the growth of cells. To visually evaluate the phototoxicity of DNPs/N@PDA in HeLa cells, the live cells were stained green by Calcein‐AM and the dead cells were stained red by propidium iodide (PI), respectively. As shown in Figure 5C and Figure S5B in the Supporting Information, almost all the cells were destroyed to death after treating by the DNPs/N@PDA + NIR. The results were consistent with those of MTT assay.

### 2.4 In Vivo Biodistribution Study

**Figure 6:**
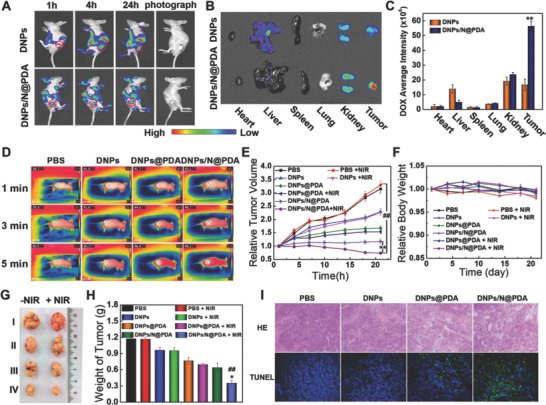
A) Time‐lapse NIR fluorescence images of HeLa tumor‐bearing nude mice. B) Ex vivo NIR fluorescence images of tumors and major organs. C) Average fluorescence signals of DOX in tumors and major organs were quantified at 24 h post tail‐vein injection with DNPs and DNPs/N@PDA. The data were expressed as mean ± SD (*n* = 3). (**p < 0.01 versus DNPs group). D) Infrared thermal images of HeLa‐tumor bearing nude mice's right hind limb after NIR irradiation (808 nm, 5 W cm^−2^, 5 min). E) Relative tumor volume of the mice treated by PBS, DNPs, DNPs@PDA, and DNPs/N@PDA with or without NIR irradiation (808 nm, 5 W cm^−2^, 5 min) (**p < 0.01 versus DNPs/N@PDA group without NIR irradiation and ^##^ *p* < 0.01 versus PBS group treated by NIR laser). F) Relative body weight of the mice. G) Representative photos of excised tumors 21 d after treatments. H) Average tumor weight at 21st day of posttreatments (*p < 0.05 versus DNPs/N@PDA group without NIR irradiation and ^##^ *p* < 0.01 versus PBS group treated by NIR laser). I) Histological staining (H&E) and terminal‐deoxynucleoitidyl transferase mediated nick end labeling (TUNEL) staining (200×) of the tumor tissues after injection of PBS, DNPs, DNPs@PDA, and DNPs/N@PDA for 24 h treated by NIR laser for 5 min (808 nm, 5 W cm^−2^). (*n* = 5, mean ± SD).

To monitor the biodistribution of DOX in the mice, DNPs, DNPs@PDA, and DNPs/N@PDA were intravenously injected into HeLa tumor‐bearing nude mice with an identical DOX dosage of 3 mg kg^−1^. As shown in **Figure** **6**A, it was observed that at 4 h post injection, the intense fluorescence could be detected in the liver and kidney but no obvious fluorescence in the tumor sites (the red circle) in the DNPs‐treated groups, and the signal intensity of DOX‐loaded DNPs in liver and kidney was stronger than that of DOX‐loaded DNPs/N@PDA. After 24 h post injection, the signal intensity of liver and lung began to wane for both DNPs treatment group and DNPs/N@PDA treatment group, indicating the quick clearance and interception by the liver and kidney. While, the tumor fluorescence signal of group treated by DNPs/N@PDA increased due to the long‐term circulation of PDA coated nanoparticles. After 24 h post injection, the ex vivo imaging toward major organs (heart, liver, spleen, lung, and kidney) and tumors were performed (Figure 6B). In the DNPs/N@PDA group, the tumor exhibited much stronger fluorescence compared with other tissues, indicating that DNPs/N@PDA could improve the concentration and extend the dwell time of DOX at the site of tumor. In contrast, a large fraction of DOX was accumulated into the liver and kidney in the controlled group intravenously injected with DNPs. It turned out that the DOX diffused out of the DNPs in the process of blood circulation, leading to the quick clearance from the body. The higher fluorescent signals of DOX in the tumors for DNPs/N@PDA group implied that this nanosystem was much more stable during blood circulation due to the shielding of PDA film. Quantitative analysis of fluorescence intensity in HeLa tumors also indicated that the intratumor content of DOX in DNPs/N@PDA‐treated group was 3.4‐fold higher than that in the DNPs‐treated groups (Figure 6C).

### 2.5 In Vivo Anticancer Activities

To confirm that the “boom‐like” drug release of DOX from DNPs/N@PDA and the enhanced tumor accumulation could achieve synergistic chemo‐/photothermal therapy in vivo, we carried out the in vivo antitumor experiments of DNPs/N@PDA. First, the photothermal effect of DNPs/N@PDA in vivo was measured. As shown in Figure 6D, after intravenous injection with 300 µL of PBS, DNPs, DNPs@PDA, and DNPs/N@PDA (both containing 3 mg kg^−1^ DOX) for 24 h, the temperature increase in the tumor region during NIR laser irradiation was recorded. For the groups treated by PBS and DNPs, the temperature in the tumor region only increased to 40.5 and 40.7 °C after irradiation for 5 min, respectively. This temperature was not high enough to destroy the tumors since the temperature above 43 °C was reported to contribute to the selective destruction of the tumor cells.^41^, ^42^ Possibly because of the passive targeting induced by the enhanced permeability and retention (EPR) effect, DNPs@PDA and DNPs/N@PDA achieved high accumulation in tumor and a maximum temperature increase up to 46.4 and 48.2 °C, respectively, much higher than 43 °C. This could cause irreversible tumor damage. To further determine the photothermal ablation effect of DNPs/N@PDA in vivo, HeLa tumor tissues treated respectively by PBS, DNPs, DNPs@PDA, and DNPs/N@PDA for 24 h with NIR irradiation for 5 min were detected by terminal‐deoxynucleoitidyl transferase mediated nick end labeling (TUNEL) apoptosis Assay Kit and stained with hematoxylin and eosin (H&E) (Figure 6I). Compared with the tumors treated by DNPs@PDA, the groups treated by DNPs/N@PDA plus NIR irradiation showed typical features of thermal damage in tumor tissues. The results of TUNEL assay were in accordance with that of H&E where a large number of apoptotic cells were labeled green in the DNPs/N@PDA plus NIR irradiation group, demonstrating the most significant antitumor activity of DNPs/N@PDA under NIR laser irradiation.

The in vivo antitumor efficacy of DNPs/N@PDA was further investigated in nude mice with HeLa cell xenograft tumor model. There was no obvious variation in mice weight in all the treated groups, suggesting that the experiment treatments were well tolerated (Figure 6F). As far as the index of tumor volume was concerned, there were no signs of antitumor effects in the groups treated with PBS with or without NIR laser irradiation, suggesting that the NIR irradiation has no effect on tumor therapy. As expected, both DOX‐containing nanoformulation and PDA‐containing formulation plus NIR laser significantly inhibited tumor growth of mice compared with PBS group. But, there was no difference in terms of antitumor effect of DNPs before and after the NIR irradiation, reconfirming the superiority of PDA film. Of note, the best performance was achieved in the group receiving DNPs/N@PDA formulation with NIR irradiation, where the tumor volume shrank remarkably along with time. On the contrary, for the other treatment, a different degree of tumor expansion was still observed. The tumor size of the group treated with DNPs/N@PDA formulation under NIR irradiation declined continuously to 0.7‐fold its original volume after 21 d of treatment, whereas the tumor sizes of the groups treated with blank PBS solution, DNPs, and DNPs@PDA were increased to around 3.3‐, 2.3‐, and 1.5‐fold the original volume, respectively (Figure 6E,G,H). All the results demonstrated that the passive targeting to tumor tissues, pH, and photothermal‐triggered fast release of DOX in the tumors as well as the local hyperthermia ablation effect of PDA contributed to an excellent antitumor activity of the DNPs/N@PDA.

**Figure 7:**
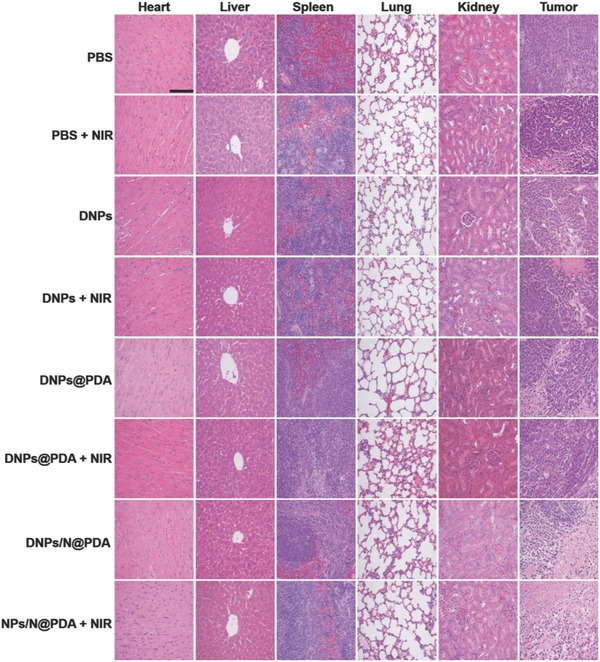
H&E staining images of tumor tissue and other major organs including heart, liver, spleen, lung, and kidney after the mice were sacrificed at 21st day post intravenous injection with PBS, DNPs, DNPs@PDA, and DNPs/N@PDA with or without NIR irradiation. DOX dosage was fixed at 3 mg kg^−1^. Magnification 200×. Scale bar: 100 µm.

On day 21, all mice were sacrificed since the tumors in control groups were too large, and the tumors were excised and weighed. Furthermore, H&E was used to stain HeLa tumor tissues and various major organs to visually evaluate the side effects. **Figure** **7** showed that the heart, liver, spleen, lung, and kidneys of various group remained the normal physiological morphologies, and no pathological changes were observed. Taken together, the carrier‐free DNPs/N@PDA combining high loading efficiency drugs and photothermal effect achieved a superior chemo‐thermotherapy of cancers with a minimal side effect in vivo.

## 3 Conclusion

In summary, we reported a simple strategy to fabricate a novel PDA‐coated NIR‐responsive “bomb‐like” carrier‐free nanosystem (DNPs/N@PDA). By using PDA self‐polymerization, we prepared stable and uniform DNPs‐based nanoparticles with thermal responsiveness, long blood circulation time, and in situ NIR‐induced rapid drug release behavior. This DNPs/N@PDA nanosystem demonstrated a high DOX loading capacity (as high as 85.8%) without any inert carrier. With NIR laser irradiation, CO_2_ and NH_3_ gases produced from the encapsulated ammonium bicarbonate for the heat generated by the coated PDA films facilitated the breakup of the PDA film outside the DNPs and concurrent the in situ release of DOX in the tumor tissue, thus leading to an enhanced chemo‐/photothermal therapy efficacy. The NIR‐responsive “bomb‐like” carrier‐free DNPs/N@PDA nanoparticles demonstrated superior in vitro and in vivo efficacy against the tumor cells. All in all, the present study illustrates the great potential of the NIR‐responsive carrier‐free nanoparticles for in situ fast DOX release to achieve augmented cancer therapy. In light of the multichoice of chemotherapeutic drugs and easy introduction of other functional modules into the surface of the PDA film for the existence of functional groups of catechol and amine, this work opens up a new avenue to tailor precise theragnostic nanosystems with high drug loading efficiency and high accumulation tumor concentration for a specific patient or disease.

## 4 Experimental Section

*Materials*: Doxorubicin hydrochloride (DOX•HCl, >98%) was purchased from Meilun Bio, China. Dopamine hydrochloride (>99%) and ammonium bicarbonate (NH_4_HCO_3_) were obtained from Alfa. DMSO, trimethylamine (TEA), Hoechst 33342, and penicillin–streptomycin were purchased from Sigma. Dulbecco's modified eagle medium, Dulbecco's phosphate buffered saline (PBS), and fetal bovine serum were purchased from Gibco. MTT was purchased from Fluka (Buchs, Switzerland). LIVE/DEAD Viability/Cytotoxicity Kit was obtained from Beyotime Institute of Biotechnology. Ultrapure water was used to prepare solutions. All the other reagents were analytical grade and used without further purification.

*Preparation of NIR‐Responsive Carrier‐Free “Nanobomb” (DNPs/N@PDA)*: These nanosystems were synthesized according to the literature with a minor modification.^16^, ^19^ First, 10 mg DOX•HCl was dissolved into 10 mL DMSO solution, followed with the addition of 1 mL of TEA. Then the mixture solution was stirred at room temperature overnight and the hydrophilic DOX•HCl molecules were converted to hydrophobic DOX molecules. After that, 200 µL of the as‐prepared DOX–DMSO solution was added dropwise into 10 mL of deionized water under vigorous stirring at 1000 rpm for 5 min and carrier‐free nanoparticles (DNPs) were prepared. Next, 0.6 mg NH_4_HCO_3_ was added into the 10 mL of 0.2 m PBS (pH 8.5) which contained 1.2 mg of dopamine and 0.1 mg of DNPs. The mixture was shaken at room temperature (25 °C) overnight. The product was further purified by water‐phase filter (220 nm). Similarly, the PDA coated DNPs nanoparticles (DNPs@PDA) were prepared by adding 1.2 mg of dopamine to 10 mL of 0.2 m PBS (pH 8.5) containing 0.1 mg of DNPs. After shaking at room temperature (25 °C) overnight, the product was further purified by water‐phase filter (220 nm).

*In Vitro Photothermal Effects*: The thermal profiles of DNPs, DNPs@PDA, and DNPs/N@PDA were measured upon irradiation of an 808 nm laser (BWT Beijing Ltd.) at 5 W cm^−2^ for 8 min with the concentration of 0.1 mg mL^−1^, and the temperature at different time points were recorded by an infrared thermal imaging camera (FLIR E8, USA). The PBS irradiated at the same conditions was used as a control.

*In Vivo Photothermal Ablation of Tumor*: The mice bearing HeLa tumor were intravenously injected with PBS, DNPs, DNPs@PDA, and DNPs/N@PDA at a DOX dosage of 3 mg kg^−1^, respectively. After 24 h, the tumors were exposed to NIR laser of 808 nm at a power density of 5 W cm^−2^ for 5 min. The photothermal images and the temperatures at different points were recorded with a thermal infrared imaging camera. After that, all the mice were sacrificed and the tumors were dissected. TUNEL apoptosis assay and H&E staining were further performed to evaluate the photothermal therapy efficiency. The mice treated by the same volume of DNPs and PBS were used as control groups. Animal care and handing procedures agreed with the guidelines evaluated and approved by ethics committee of Chinese Academy of Medical Sciences and Peking Union Medical College Institute of Biomedical Engineering.

*In Vivo Antitumor Effect*: BALB/c mice bearing tumor were subcutaneously injected with 100 µL of HeLa cells (2 × 10^6^ cells) into the right hind limbs. When the volume of the tumor xenograft reached around 100–150 mm^3^, the mice were randomly divided into eight groups (*n* = 5) and the prepared formulations (PBS, DNPs, DNPs@PDA, and DNPs/N@PDA) were injected intravenously via tail vein every 3 d for three times. The dose amount for DOX was fixed at 3 mg kg^−1^. In order to realize photothermal treatment, the tumors were treated by NIR laser irradiation (808 nm, 5 W cm^−2^) for 5 min at 1, 4, and 7 d, respectively. The caliper was used to measure the perpendicular diameter of the tumors to monitor the tumor growth. The tumor volume was calculated using the following equation: *V*(mm^3^) = *W* ^2^ *L*/2, where *W* and *L* are the shortest and longest diameters, respectively. The relative volume was calculated as *V*/*V*_0_, where *V* and *V*_0_ are the tumor volume before and after treatment, respectively. The mice weight was also recorded. After 21 d, the mice were sacrificed and their tumors were immediately harvested and photographed. The major organs were fixed by 4% formalin, embedded in paraffin, and then sectioned with 5 µm thickness for histological examinations by H&E staining. The stained slices were imaged with microscope (CKX41, Olympus, Japan) at 200 × magnifications.

## Conflict of Interest

The authors declare no conflict of interest.

## Supporting information

Supplementary

## References

[1] S. Mura , J. Nicolas , P. Couvreur , Nat. Mater. 2013, 12, 991. 24150417 10.1038/nmat3776

[2] D. Peer , J. M. Karp , S. Hong , O. C. Farokhzad , R. Margalit , R. Langer , Nat. Nanotechnology 2007, 2, 751. 10.1038/nnano.2007.387 18654426

[3] W. C. Chen , Y. Y. Yuan , D. Cheng , J. F. Chen , L. Wang , X. T. Shuai , Small 2014, 10, 2678. 24668891 10.1002/smll.201303951

[4] C. Y. Ang , S. Y. Tan , C. Teh , J. M. Lee , M. F. E. Wong , Q. Y. Qu , L. Q. Poh , M. H. Li , Y. Y. Zhang , V. Korzh , Y. L. Zhao , Small 2017, 13, 1602379. 10.1002/smll.201602379 27918645

[5] B. Feng , F. Y. Zhou , Z. A. Xu , T. T. Wang , D. G. Wang , J. P. Liu , Y. L. Fu , Q. Yin , Z. W. Zhang , H. J. Yu , Y. P. Li , Adv. Funct. Mater. 2016, 26, 7431.

[6] Y. Yi , H. J. Wang , X. W. Wang , Q. L. Liu , M. Ye , W. H. Tan , ACS. Appl. Mater. Interfaces 2017, 9, 5847. 28124556 10.1021/acsami.6b15414

[7] L. L. Feng , S. L. Gai , F. He , Y. L. Dai , C. N. Zhong , P. P. Yang , J. Lin , Biomaterials 2017, 147, 39. 28926732 10.1016/j.biomaterials.2017.09.011

[8] H. H. Fan , G. B. Yan , Z. L. Zhao , X. X. Hu , W. H. Zhang , H. Liu , X. Y. Fu , T. Fu , X. B. Zhang , W. H. Tan , Angew. Chem., Int. Ed. 2016, 128, 5567.

[9] F. Li , T. Y. Li , W. Cao , L. Wang , H. P. Xu , Biomaterials 2017, 133, 208. 28441615 10.1016/j.biomaterials.2017.04.032

[10] W. Cheng , J. P. Nie , N. S. Gao , G. Liu , W. Tao , X. J. Xiao , L. J. Jiang , Z. G. Liu , X. W. Zeng , L. Mei , Adv. Funct. Mater. 2017, 27, 1704135.

[11] X. D. Xu , P. E. Saw , W. Tao , Y. J. Li , X. Y. Ji , M. Yu , M. Mahmoudi , J. Rasmussen , D. Ayyash , Y. X. Zhou , O. C. Farokhzad , J. J. Shi , Nano Lett. 2017, 17, 4427. 28636389 10.1021/acs.nanolett.7b01571 PMC5615408

[12] J. F. Zhang , Y. C. Liang , X. D. Lin , X. Y. Zhu , L. Yan , S. L. Li , X. Yang , G. Y. Zhu , A. L. Rogach , P. K. N. Yu , P. Shi , L. C. Tu , C. C. Chang , X. H. Zhang , X. F. Chen , W. J. Zhang , C. S. Lee , ACS Nano 2015, 9, 9741. 26390118 10.1021/acsnano.5b02513

[13] P. Fattahi , A. Borhan , M. R. Abidian , Adv. Mater. 2013, 25, 4555. 23824544 10.1002/adma.201301033

[14] P. Fattahi , A. Borhan , M. R. Abidian , 6th Int. IEEE/EMBS Conf. on Neural Engineering (NER), San Diego, CA, November 2013.

[15] M. Antensteiner , M. Khorrami , F. Fallahianbijan , A. Borhan , M. R. Abidian , Adv. Mater. 2017, 29, 1702576. 10.1002/adma.201702576 PMC5798879 28833611

[16] C. T. Yu , M. J. Zhou , X. J. Zhang , W. J. Wei , X. F. Chen , X. H. Zhang , Nanoscale 2015, 7, 5683. 25740312 10.1039/c5nr00290g

[17] J. F. Zhang , S. L. Li , F. F. An , J. Liu , S. B. Jin , J. C. Zhang , P. C. Wang , X. H. Zhang , C. S. Lee , X. J. Liang , Nanoscale 2015, 7, 13503. 26199064 10.1039/c5nr03259h PMC4636738

[18] H. Lee , S. M. Dellatore , W. M. Miller , P. B. Messersmith , Science 2007, 318, 426. 17947576 10.1126/science.1147241 PMC2601629

[19] L. S. Lin , Z. X. Cong , J. B. Cao , K. M. Ke , Q. L. Peng , J. H. Gao , H. H. Yang , G. Liu , X. Y. Chen , ACS Nano 2014, 8, 3876. 24654734 10.1021/nn500722y PMC4564054

[20] W. Cui , M. Z. Li , J. Y. Liu , B. Wang , C. Zhang , L. Jiang , Q. F. Cheng , ACS Nano 2014, 8, 9511. 25106494 10.1021/nn503755c

[21] J. Y. Park , T. F. Brust , H. J. Lee , S. C. Lee , V. J. Watts , Y. Yeo , ACS Nano 2014, 8, 3347. 24628245 10.1021/nn405809c PMC4107448

[22] Y. P. Ding , S. S. Su , R. R. Zhang , L. H. Shao , Y. L. Zhang , B. Wang , Y. Y. Li , L. Chen , Q. Yu , Y. Wu , G. J. Nie , Biomaterials 2017, 113, 243. 27829203 10.1016/j.biomaterials.2016.10.053

[23] Y. Y. Li , C. H. Jiang , D. W. Zhang , Y. Wang , X. Y. Ren , K. L. Ai , X. S. Chen , L. H. Lu , Acta Biomater. 2017, 47, 124. 27721008 10.1016/j.actbio.2016.10.010

[24] Y. Chen , K. L. Ai , J. H. Liu , X. Y. Ren , C. H. Jiang , L. H. Lu , Biomaterials 2015, 77, 198. 26606445 10.1016/j.biomaterials.2015.11.010

[25] K. J. Chen , H. F. Liang , H. L. Chen , Y. C. Wang , P. Y. Cheng , H. L. Liu , Y. N. Xia , H. W. Sung , ACS Nano 2013, 7, 438. 23240550 10.1021/nn304474j

[26] F. F. Zhao , J. Zhou , X. J. Su , Y. H. Wang , X. S. Yan , S. N. Jia , B. Du , Small 2017, 13, 1603990.

[27] E. Y. Chuang , C. C. Lin , K. J. Chen , D. H. Wan , K. J. Lin , Y. C. Ho , P. Y. Lin , H. W. Sung , Biomaterials 2016, 93, 48. 27070992 10.1016/j.biomaterials.2016.03.040

[28] M. Yu , F. Guo , F. P. Tan , N. Li , J. Controlled Release 2015, 215, 91. 10.1016/j.jconrel.2015.08.003 26256259

[29] K. J. Chen , E. Y. Chaung , S. P. Wey , K. J. Lin , F. Cheng , C. C. Lin , H. L. Liu , H. W. Tseng , C. P. Liu , M. C. Wei , C. M. Liu , H. W. Sung , ACS Nano 2014, 8, 5105. 24742221 10.1021/nn501162x

[30] J. Z. Xia , G. Feng , X. R. Xia , L. Hao , Z. G. Wang , Int. J. Nanomed. 2017, 12, 1803. 10.2147/IJN.S113366 PMC5345996 28293107

[31] H. S. Min , S. Son , D. G. You , T. W. Lee , J. Lee , S. Lee , J. Y. Yhee , J. Lee , M. H. Han , J. H. Park , S. H. Kim , K. Choi , K. Park , K. Kim , L. C. Kwon , Biomaterials 2016, 108, 57. 27619240 10.1016/j.biomaterials.2016.08.049

[32] Y. L. Liu , K. L. Ai , J. H. Liu , M. Deng , Y. Y. He , L. H. Lu , Adv. Mater. 2013, 25, 1353. 23280690 10.1002/adma.201204683

[33] Y. X. Xing , J. X. Zhang , F. Chen , J. J. Liu , K. Y. Cai , Nanoscale 2017, 9, 8781. 28621774 10.1039/c7nr01857f

[34] H. J. Li , J. Z. Du , J. Liu , X. J. Du , S. Shen , Y. H. Zhu , X. Y. Wang , X. D. Ye , S. M. Nie , J. Wang , ACS Nano 2016, 10, 6753. 27244096 10.1021/acsnano.6b02326

[35] H. J. Li , J. Z. Du , X. J. Du , C. F. Xu , C. Y. Sun , H. X. Wang , Z. T. Cao , X. Z. Yang , Y. H. Zhu , S. Nie , J. Wang , Proc. Natl. Acad. Sci. USA 2016, 113, 4164. 27035960 10.1073/pnas.1522080113 PMC4839420

[36] W. J. Jiang , F. Mo , X. Jin , L. Chen , L. J. Xu , L. Q. Guo , F. F. Fu , Adv. Mater. Interfaces 2017, 4, 1700425.

[37] Q. S. Zheng , T. R. Lin , H. Y. Wu , L. Q. Guo , P. R. Ye , Y. L. Hao , Q. Q. Guo , J. Z. Jiang , F. F. Fu , G. N. Chen , Int. J. Pharm. 2014, 463, 22. 24393764 10.1016/j.ijpharm.2013.12.045

[38] L. Ding , X. B. Zhu , Y. L. Wang , B. Y. Shi , X. Ling , H. J. Chen , W. H. Nan , A. Barrett , Z. L. Guo , W. Tao , J. Wu , X. J. Shi , Nano Lett. 2017, 17, 6790. 29058908 10.1021/acs.nanolett.7b03021

[39] Q. Liu , X. M. Chen , J. L. Jia , W. F. Zhang , T. Y. Yang , L. Y. Wang , G. H. Ma , ACS Nano 2015, 9, 4925. 25898266 10.1021/nn5066793

[40] J. Z. Xia , G. Feng , X. R. Xia , L. Hao , Z. G. Wang , Int. J. Nanomed. 2017, 12, 1803. 10.2147/IJN.S113366 PMC5345996 28293107

[41] M. B. Zheng , C. X. Yue , Y. F. Ma , P. Gong , P. F. Zhao , C. F. Zheng , Z. H. Sheng , P. F. Zhang , Z. H. Wang , L. T. Cai , ACS Nano 2013, 7, 2056. 23413798 10.1021/nn400334y

[42] Y. Tang , T. J. Lei , R. Manchanda , A. Nagesetti , A. F. Fernandez , S. Srinivasan , A. J. McGoron , Pharm. Research 2010, 27, 2242. 10.1007/s11095-010-0231-6 20694526

