# Supplementary material for: NIR‐Activated Polydopamine‐Coated Carrier‐Free “Nanobomb” for In Situ On‐Demand Drug Release
Source: Adv Sci (Weinh). 2018 May 9;5(7):1800155. doi: 10.1002/advs.201800155 (PMC6051140; doi:10.1002/advs.201800155)
Supplement: Supplementary file 1 — Supplementary [file ADVS-5-1800155-s001.pdf]

Copyright WILEY-VCH Verlag GmbH & Co. KGaA, 69469 Weinheim, Germany, 2018.

ADVANCED SCIENCE Open Access

Supporting Information

for *Adv. Sci.,* DOI: 10.1002/advs.201800155

NIR-Activated Polydopamine-Coated Carrier-Free "Nanobomb" for In Situ On-Demand Drug Release

Minghui Li, Xuetan Sun, Ning Zhang, Wei Wang, Yang Yang, Huizhen Jia, and Wenguang Liu**

Supporting Information

NIR-Activated Polydopamine-Coated Carrier-free "Nanobomb" for In Situ On-demand Drug Release

Minghui Li[1], Xuetan Sun[1], Ning Zhang[1], Wei Wang[1], Yang Yang[1], Huizhen Jia[1,2,], Wenguang Liu[1,]**

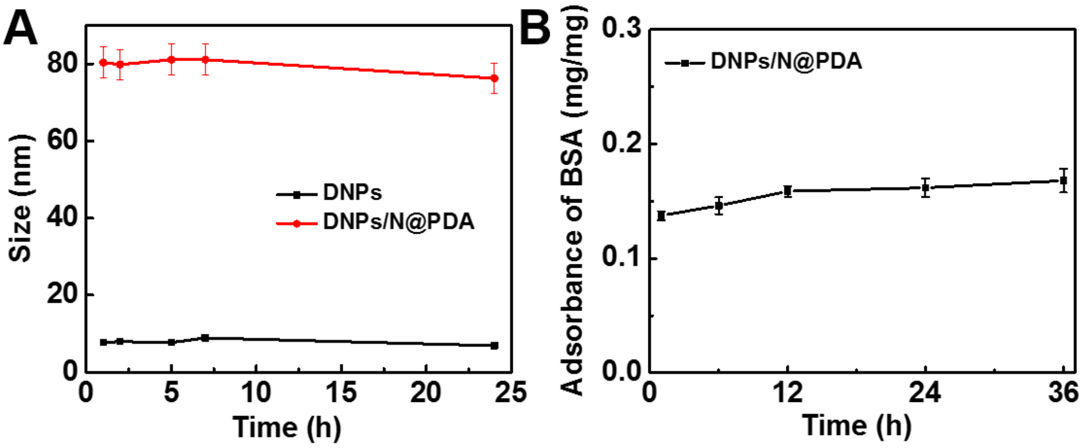

Figure S1. A) Variation in the hydrodynamic diameters of DNPs and DNPs/N@PDA as a function of time in 10% serum-containing PBS buffer solution; B) Protein adsorption of DNPs/N@PDA at different times. 1 mL of DNPs/N@PDA solution (1 mg/mL) was added to 1 mL BSA solution (1 mg/mL) and co-incubated with shaking at 37 °C for 0.5 h prior to measurement.

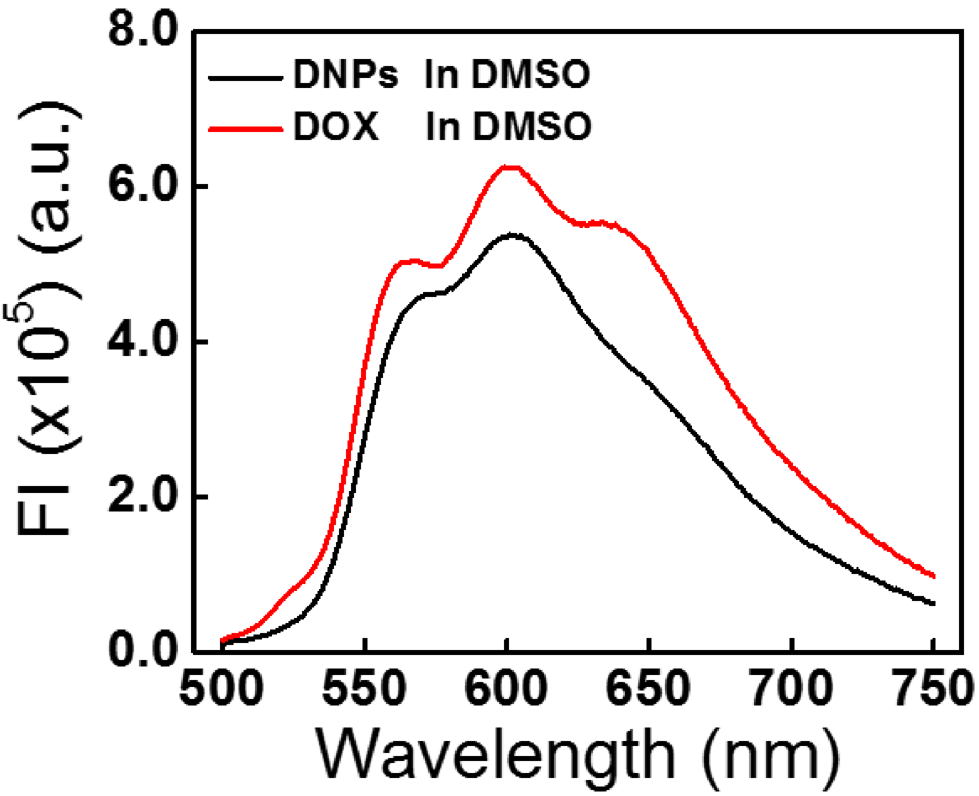

Figure S2. Fluorescence spectra of DOX and DNPs in DMSO solution with the excitation wavelength of 488 nm.

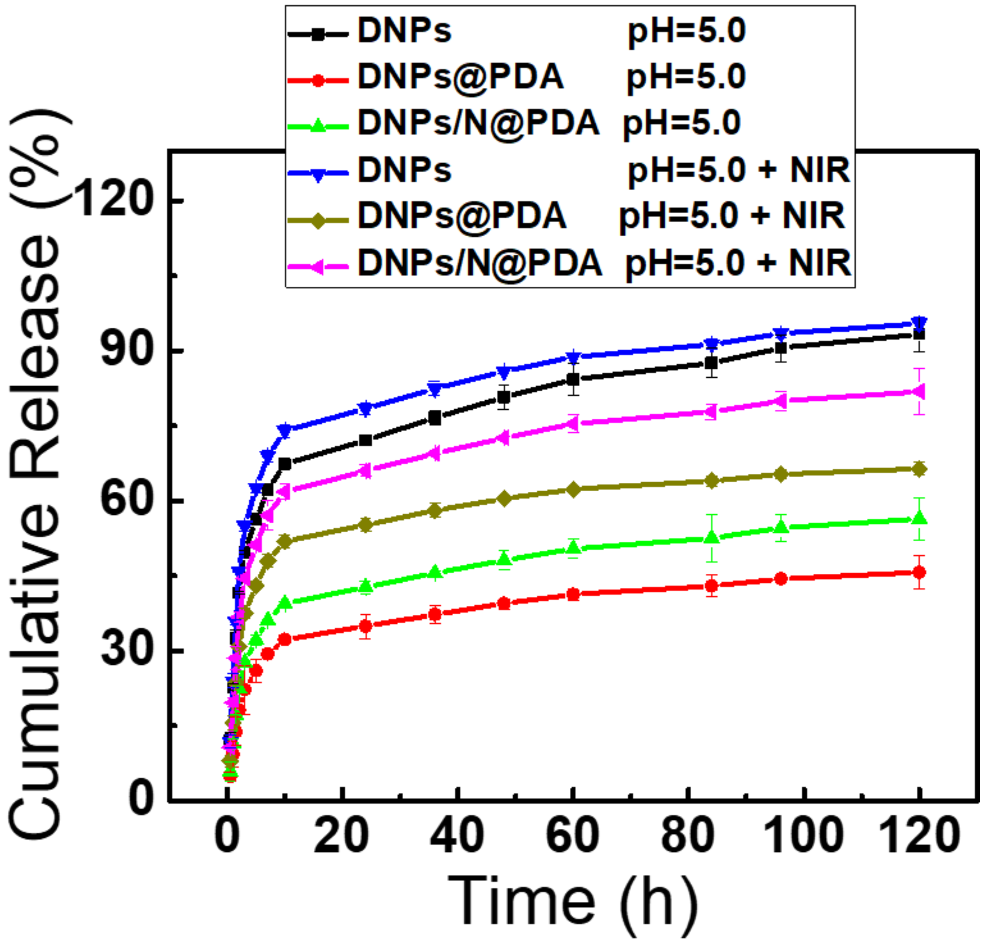

Figure S3. Cumulative release profiles of DOX from DNPs, DNPs@PDA and DNPs/N@PDA in pH 5.0 PBS buffer solution with or without NIR irradiation (808 nm, 5 W cm^{-2}, 5 min).

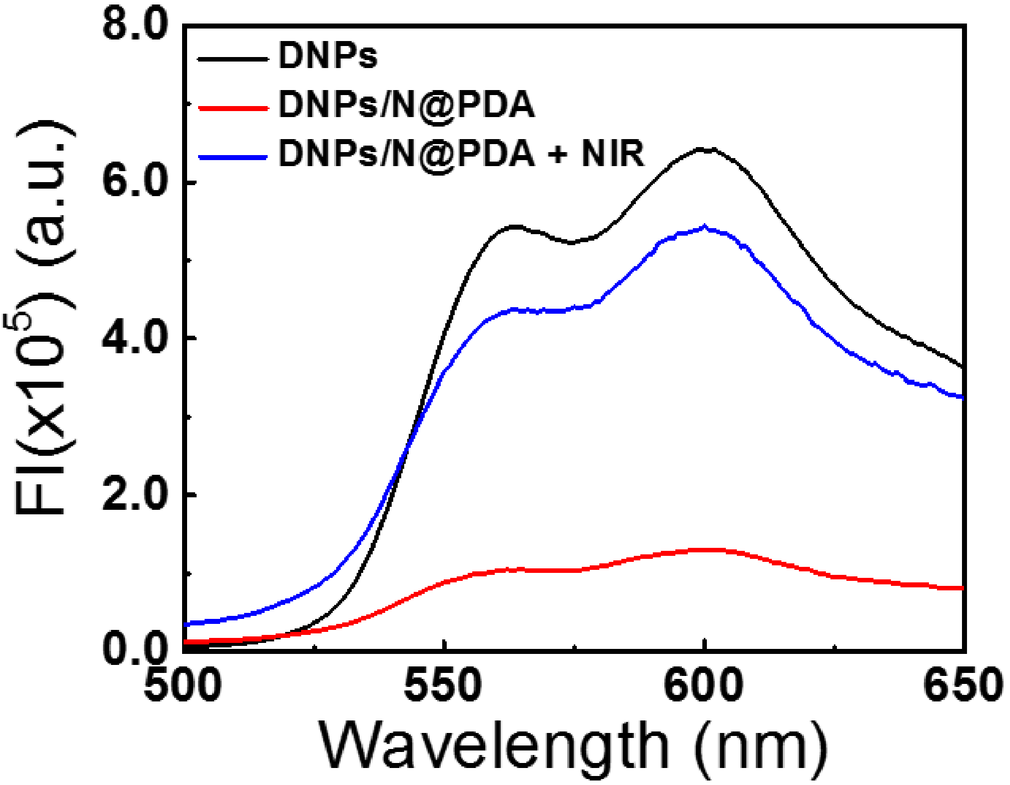

Figure S4. Fluorescence spectra of DNPs, DNPs/N@PDA with/without NIR irradiation (808 nm, 5 W cm^{-2}, 5 min).

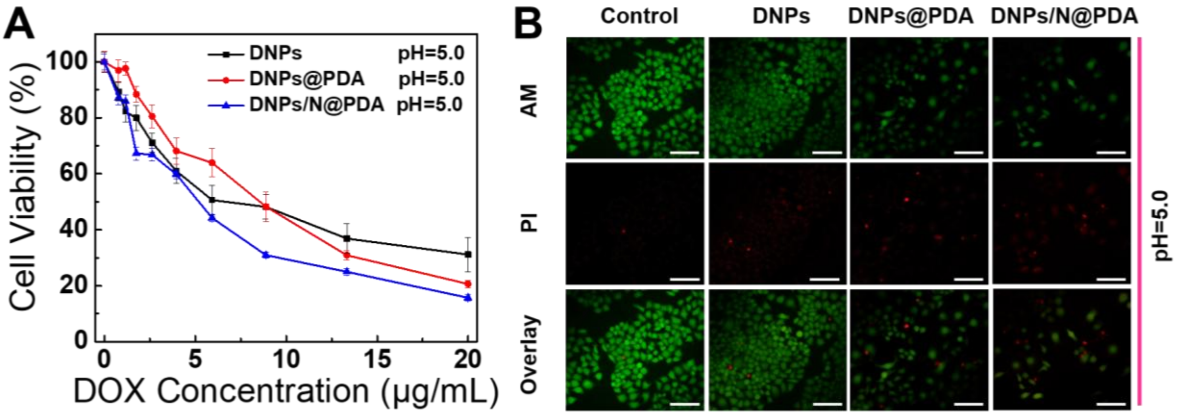

Figure S5. (A) *In vitro* cytotoxicity of DNPs, DNPs@PDA and DNPs/N@PDA in pH 5.0 medium at different DOX concentrations against HeLa cells after 48 h incubation; (B) Confocal fluorescence microscopy images of HeLa cells incubated in pH 5.0 medium co-stained with Calcein-AM (green, live cells) and propidium iodide (PI) (red, dead cells). Scale bars: 100 μm.

Experimental Section

1. Cell culture

The human cervix carcinoma (HeLa) cells were grown in DMEM medium with 10% fetal bovine serum and 1% antibiotics (penicillin-streptomycin, 10000 U mL^{-1}) at 37 ^{o}C in a humidified atmosphere of 5% CO_2.

2. TEM observation

The morphologies of the fabricated DNPs/N@PDA dispersed on a carbon-coated copper grid were investigated using a transmission electron microscope (TEM) (JEOL JEM-2100F, Japan) with an acceleration voltage of 80 kV. Before visualization, a droplet of the sample solution was placed on a copper grid with formvar film.

3. Particle size and zeta potential measurement

Dynamic light scattering (DLS) was used to determine the hydrodynamic size and zeta potential of the nanoparticles at 25 ^{o}C by Nano-ZSZEN3600 (Malvern Instruments). Data were shown as ± standard deviation (SD) based on three independent measurements.

4. Protein adsorption assay

Protein binding was assayed by mixing the DNPs/N@PDA solution (1 mg mL^{-1}) and the BSA solution (1 mg mL^{-1}) in centrifuge tubes and shaking for 30 min at 37°C. After centrifugation the UV absorbance of the supernatant was determined at 280 nm. The BSA concentration was obtained using a standard calibration curve. The protein adsorbed on the DNPs/N@PDA was calculated based on the following equation: $BSA_{(adsorbed)}=([BSA]_i-[BSA]_s)$ /m. Where $[BSA]_i$ and $[BSA]_s$ refers to the initial weight of BSA and the BSA weight in the supernatant after centrifugation, respectively; m is the weight of the DNPs/N@PDA added into the solution.

5. Fluorescence spectra and XRD patterns of the nanoparticles

The fluorescence spectra of the nanoparticles were obtained by Fluorescence spectrometer (Edinburgh F920, UK). XRD patterns were obtained using an X-ray diffractometer (Brooker, D8 Advanced, Germany) to confirm the NH_4HCO_3 was successfully encapsulated.

6. Determination of drug loading efficiency (DLE)

The Fluorescence intensity of DNPs dispersion in DMSO solution (FI (DNPs)) was measured after separation by centrifugation. The fluorescence intensity of DOX in DMSO solution (FI (DOX)) was also measured. By comparison, the DLE was calculated based on the formula: DLE% = FI (DNPs) / FI (DOX) × 100%. The fluorescence absorbance intensity of DOX was measured at 600 nm by a F920 fluorescence spectrometer (Edinburgh) with excitation wavelength of 488 nm.

7. *In vitro* drug release test

The release profiles of DOX from DNPs, DNPs@PDA and DNPs/N@PDA were determined by dialysis method at 37 ^{o}C in different conditions: (a) phosphate buffer, pH=7.4; (b) phosphate buffer, pH=5.0 (without NIR laser); (c) phosphate buffer, pH=5.0 (with NIR laser, 5 W cm^{-2} for 5 min). Briefly, 3 mL of the DNPs, DNPs@PDA or DNPs/N@PDA solution was transferred into a dialysis tube (MWCO: 3500 Da, Shanghai green bird science and technology development Co. LTD) followed by immersion in 5 mL of corresponding PBS at 37 ^{o}C with constant shaking at 200 rpm. The ionic strength of all the buffer solution was fixed at 0.2 M. At pre-determined time points, 5 mL of the PBS was collected and an equal volume of fresh buffer solution was added. Then the concentrations of the DOX released from agents were measured on the basis of the fluorescence absorbance intensity at 600 nm, using a standard calibration curve obtained.

8. *In vitro* cytotoxicity

The *in vitro* cytotoxicity of DNPs/N@PDA against HeLa cells was assessed by the standard MTT assay. First, HeLa cells were seeded into a 96-well plate at a density of 6.0×10^{3} cells/well and then incubated in 100 μL DMEM containing 10% FBS for 24 h at 37 ^{o}C prior to samples addition. After the cells were incubated with different concentration of DNPs, DNPs@PDA and DNPs/N@PDA for 4 h, the medium was removed and the cells were further incubated in the fresh DMEM for 48 h. To investigate the photocytotoxicity of the

formulations, the cells co-incubated with the samples at pH 5.0 were treated with NIR irradiation (808 nm, 5 W cm^{-2}, 1 min) while other operations were the identical unless noted. After that, the medium was removed and 200 μL of fresh DMEM containing 10% FBS and 20 μL of MTT (5 mg/mL in PBS buffer solution) was added and the cells were further incubated for 4 h at 37 ^oC. Subsequently, the medium was replaced by 150 μL of DMSO. The absorbance intensity at 570 nm was measured using a microplate reader (Bio-Red, Model 550, USA). The relative cell viability was calculated as:

Cell viability (%) = (OD(sample)/OD(control)) × 100%

Where OD(control) and OD(sample) were obtained in the absence and presence of the samples respectively. Data were expressed as mean ± standard deviation (SD) based on three independent measurements.

For the photothermal ablation assay, the HeLa cells were treated with the same method as mentioned above. The DOX concentration was fixed at 5 μg/mL. After incubation for 48 h, the medium was replaced by fresh medium and stained with LIVE/DEAD Viability/Cytotoxicity Kit according to the directions. Finally, the cells were detected with a fluorescent microscope (OLYMPUS, Japan).

9. *In vitro* cellular internalization assay

Confocal laser scanning microcopy was used to determine the *in vitro* cellular uptake of the DNPs, DNPs@PDA and DNPs/N@PDA in HeLa cells under three different conditions: (Ⅰ) pH 7.4 (neutral environment), (Ⅱ) pH 5.0 (acid environment), (Ⅲ) pH 5.0 (acid environment) with NIR irradiation. The HeLa cells were seeded in a single dish with a density of 1.0×10^5 cells/well and incubated in 1 mL of DMEM containing 10% FBS for 24 h at 37 ^oC. Then the medium was replaced by fresh medium containing DNPs, DNPs@PDA and DNPs/N@PDA with the pH value of 7.4 or pH 5.0, respectively (the concentration of DOX was 10 μg/mL). After incubated for 4 h, the medium was removed and fresh culture medium was added, and the HeLa cells treated with the materials at pH 5.0 were exposed under laser irradiation (808

nm, 5 W cm^{-2}) for 5 min. Then the cells were further cultured for another 2 h. After removing the medium, the cells were washed with PBS for several times and 1 mL of DMEM containing 10 μL Hoechst 33342 was added and the cells were incubated for 15 min at 37 ^{o}C. Prior to the imaging under a confocal laser scanning microscopy (Nikon C1-si TE2000, Japan, excitation filter 488 nm and emission cut-off filter 570-630 nm for red light), the cells were washed several times by 1 mL of PBS. It was noted that the exposure to strong light should be avoided in the whole process to protect the fluorescent dyes.

10. Flow cytometry analysis

Flow cytometry was used to quantitatively evaluate the cellular internalization of DOX. Briefly, the HeLa cells were seeded in the 24-well plates with a density of 6 × 10^{4} cells/well and incubated for 24 h. After that, the cells were treated with 1 mL of fresh DMEM containing the as-prepared samples. The cells were incubated for 4 h with/without NIR irradiation (808 nm, 5 W cm^{-2}, 5 min). Then the cells were further incubated for another 2 h, and the medium was removed. Immediately, the cells were washed by fresh PBS thrice. Then the cells were trypsinized with 0.25% trypsin for 3 min at 37 ^{o}C. Subsequently, the cells were collected, washed and resuspended in 500 μL PBS. The fluorescence intensity was detected by a flow cytometer (BD LSRFortessa, USA). The blank cells served as a negative control.

11. Animals and tumor models

Male BALB/c nude mice (5 weeks old, ca. 20 g body weight) were purchased from Chinese Academy of Medical Science & Peking Union Medical College Institute of Biomedical Engineering (Tianjin). Animal care and handing procedures agreed with the guidelines evaluated and approved by ethics committee of Chinese Academy of Medical Sciences and Peking Union Medical College Institute of Biomedical Engineering. The HeLa tumor was inoculated by subcutaneous injection of 100 μL of PBS containing 2×10^{6} cells on the right hind limb of each mouse. When the volume of tumor xenograft reached around 150 mm^{3}, the mice were dived into several groups randomly for the following experiments.

12. *In vivo* and *ex vivo* fluorescence imaging

The *in vivo* and *ex vivo* fluorescence imaging was performed in male BALB/c nude mice bearing HeLa tumor on right hind limb. The as-prepared DNPs and DNPs/N@PDA formula at a DOX dosage of 3 mg kg^{-1} were intravenously injected into the mice bearing HeLa tumor via tail vein.

For *in vivo* fluorescent imaging and biodistribution assays, the mice were anesthetized by trichloroacetaldehyde hydrate (10%) with a dosage of 40 mg/kg body weight at 1 h, 4 h and 24 h post-injection, respectively. The DOX-related fluorescence was detected by a single-filter set with the excitation wavelength of 480 nm and emission wavelength of 600 nm in the absence of autofluorescence produced by the skin and blood vessels. For *ex vivo* fluorescence imaging and biodistribution assay, after 24 h post-injection, the mice were sacrificed and the tumors and major organs were dissected, washed with cold saline and then subjected to Xtreme imaging system to obtain the fluorescence images. Tumors and organs were stored at -80 ^{o}C for further use.

13. Statistical analysis

All data were expressed as the mean ± standard deviation (S.D.) (n = 3~5). The statistical significance between different groups was evaluated with Student's t-test. $p<0.05$ was considered to be statistically significant.
